# Chronic Intermittent Ethanol Exposure Increases Ethanol Consumption Following Traumatic Stress Exposure in Mice

**DOI:** 10.3389/fnbeh.2020.00114

**Published:** 2020-06-30

**Authors:** Veronica M. Piggott, Scott C. Lloyd, Shane A. Perrine, Alana C. Conti

**Affiliations:** ^1^Research & Development Service, John D. Dingell VA Medical Center, Detroit, MI, United States; ^2^Department of Neurosurgery, Wayne State University School of Medicine, Detroit, MI, United States; ^3^Department of Psychiatry and Behavioral Neurosciences, Wayne State University School of Medicine, Detroit, MI, United States

**Keywords:** post-traumatic stress disorder, mouse single-prolonged stress, chronic intermittent ethanol, ethanol consumption, alcohol use disorder, impulsivity, anhedonia

## Abstract

Individuals with post-traumatic stress disorder (PTSD) often use alcohol to cope with their distress. This aberrant use of alcohol often develops into alcohol use disorder (AUD) leading to high rates of PTSD-AUD co-occurrence. Individuals with comorbid PTSD-AUD have more intense alcohol cravings and increased relapse rates during withdrawal than those with AUD alone. Also, individuals with PTSD or AUD alone often show similar psychological behaviors, such as impulsivity and anhedonia. Extensive clinical studies on the behavioral effects of PTSD-AUD comorbidity, namely alcohol use, have been performed. However, these effects have not been well studied or mechanistically explored in animal models. Therefore, the present study evaluated the effects of traumatic stress comorbid with alcohol exposures on ethanol intake, impulsivity, and anhedonia in mice. Adult male C57Bl/6 mice were first exposed to either mouse single-prolonged stress (mSPS), an animal model that has been validated for characteristics akin to PTSD symptoms, or control conditions. Baseline two-bottle choice ethanol consumption and preference tests were conducted after a 7-day isolation period, as part of the mSPS exposure. Next, mice were exposed to air or chronic intermittent ethanol (CIE), a vapor-induced ethanol dependence and withdrawal model, for 4 weeks. Two-bottle choice ethanol drinking was used to measure dependence-induced ethanol consumption and preference during periods intervening CIE cycles. The novelty suppressed feeding (NSF) test was used to evaluate impulsivity and anhedonia behaviors 48 h after mSPS and/or repeated CIE exposure. Results showed that, compared to control conditions, mSPS did not affect baseline ethanol consumption and preference. However, mSPS-CIE mice increased Post-CIE ethanol consumption compared to Control-Air mice. Mice exposed to mSPS had a shorter latency to feed during the NSF, whereas CIE-exposed mice consumed less palatable food reward in their home cage after the NSF. These results demonstrate that mice exposed to both mSPS and CIE are more vulnerable to ethanol withdrawal effects, and those exposed to mSPS have increased impulsivity, while CIE exposure increases anhedonia. Future studies to examine the relationship between behavioral outcomes and the molecular mechanisms in the brain after PTSD-AUD are warranted.

## Introduction

Post-traumatic stress disorder (PTSD) is a serious mental health disorder that people may develop directly or indirectly after experiencing a traumatic event(s). According to the DSM-5, PTSD symptoms include flashbacks, avoidance behavior of the traumatic event(s), negative mood, and hyperarousal (American Psychiatric Association, 2013). The lifetime prevalence of PTSD in the United States population is estimated to be 6.8% (Kessler et al., 2005). Combat-exposed military personnel and Veterans have a higher risk of developing PTSD than civilians (Hoge et al., 2006; Petrakis et al., 2016). Individuals who are diagnosed with PTSD have an increased propensity (28–85%) to develop alcohol use disorder (AUD; Kessler et al., 1995; Baker et al., 2009; Ralevski et al., 2014).

Characteristics of AUD include high tolerance to short-term effects of ethanol and vulnerability to withdrawal symptoms, such as anhedonia, during alcohol abstinence (Becker, 2008; Martinotti et al., 2008; Hatzigiakoumis et al., 2011; Pava and Woodward, 2012). Since alcohol is an effective anxiolytic, individuals with PTSD often self-medicate with alcohol to alleviate PTSD symptoms and negative emotions, which can also contribute to the development of AUD (Carter et al., 2011). Individuals with comorbid PTSD and AUD have more intense alcohol cravings and relapse more frequently during withdrawal than those with AUD only (Brown et al., 1999; Ouimette et al., 1999; Berenz et al., 2017), suggesting that the comorbid disorder is unique from either condition alone.

Clinical research has shown that PTSD symptoms such as hyperarousal and negative mood are strongly related to impulsivity (Armour et al., 2016; Contractor et al., 2016; Roley et al., 2017). Individuals with PTSD symptoms had significant disinhibition behavior compared to traumatized control individuals without PTSD symptoms (Casada and Roache, 2005). Those with PTSD also had impaired judgment in dangerous situations, especially when rewarding stimuli were involved (Casada and Roache, 2005; Roley et al., 2017).

The behavioral outcomes of PTSD-AUD comorbidity such as hyperarousal (Saladin et al., 1995) and increased alcohol relapse (Drapkin et al., 2011; Petrakis and Simpson, 2017) have been shown in clinical studies. However, drinking behavior and other psychological behaviors such as impulsivity and negative affect after traumatic stress and prolonged ethanol exposure and withdrawal have not been fully evaluated in animal models (Gilpin and Weiner, 2017). Mouse single-prolonged stress (mSPS) is based on a rodent animal model that has phenotypes akin to most behavioral and physiological symptoms in clinical PTSD (Liberzon et al., 1997, 1999; Yamamoto et al., 2009; Pitman et al., 2012; Perrine et al., 2016; Flandreau and Toth, 2018). For example, mSPS-exposed mice showed increased freezing behavior when re-exposed to an SPS-associated cue (tone) presented 7 days after mSPS (Perrine et al., 2016), which parallels the augmentation of fear responses when individuals with PTSD encounter trauma-related cues (Garfinkel et al., 2014; Gonzalez and Martinez, 2014). Also, mSPS exposure enhanced dexamethasone suppression of corticosterone levels compared to controls, demonstrating increased negative feedback sensitivity of the hypothalamus-pituitary-adrenal axis, which has been reported in humans with PTSD (Yehuda, 2009; Morris et al., 2012; Perrine et al., 2016). Using this model of traumatic stress exposure, we examined ethanol dependence behaviors using Chronic Intermittent Ethanol (CIE) vapor exposure. CIE is an animal model that has been widely used in alcohol dependence and withdrawal studies (Becker and Lopez, 2004; Becker and Ron, 2014; Anderson et al., 2016a,b; Rose et al., 2016). Repeated CIE involves an extended period of ethanol vapor exposure followed by a brief period of ethanol abstinence. This animal model has shown that mice develop tolerance to the aversive effects of ethanol during the conditioned taste aversion test, which causes escalated ethanol consumption once alcohol dependence has developed (Lopez et al., 2012). Mice exposed to CIE have high blood ethanol concentrations (BECs) that are considered to be intoxicating (Becker and Lopez, 2004). Repeated CIE exposure and ethanol abstinence also induce withdrawal symptoms such as anxiety-like behavior and anhedonia. For example, mice exposed to repeated CIE buried more marbles during the marble burying test and had a longer latency to feed during the novelty suppressed feeding (NSF) test (Rose et al., 2016; Jury et al., 2017).

Using the mSPS and CIE to model PTSD-AUD comorbidity, this study examined the effects of traumatic stress on PTSD-associated behavioral outcomes, namely ethanol intake, impulsivity, and anhedonic responses. We hypothesized that mice exposed to mSPS would demonstrate increased and sustained ethanol consumption and preference after prolonged exposure to ethanol vapor and withdrawal, which would be accompanied by increased impulsivity and anhedonia.

## Materials and Methods

### Animals

Male C57Bl/6 mice (*n* = 45) were bred in-house at Wayne State University (WSU) facilities. Mice were housed on a 12-h light/dark cycle (lights on at 6 am) in groups of 2–5 in standard microisolator polycarbonate cages under controlled temperature (21–24°C) and humidity (30–40%) with *ad libitum* access to food and water, except during the mSPS procedure and NSF testing, when food, but not water, was restricted. All procedures were approved by the WSU Institutional Animal Care and Use Committee. All experimental procedures were conducted according to the National Institute of Health Office of Laboratory Animal Welfare for the Care and Use of Laboratory Animals at the WSU Division of Laboratory Animal Resources facilities, which are accredited by the Association for Assessment and Accreditation of Laboratory Animal Care. Data from the first cohort of mice (*n* = 28) were used for the baseline limited access two-bottle choice ethanol drinking test. Data from the second cohort of mice (*n* = 18) were used for the Post-CIE limited access two-bottle choice ethanol drinking test. Data from the third cohort of mice (*n* = 27) were used for the NSF test. All cohorts of mice were exposed to mSPS, CIE, and two-bottle choice ethanol drinking.

### Mouse Single-Prolonged Stress (mSPS)

**Figure 1:**
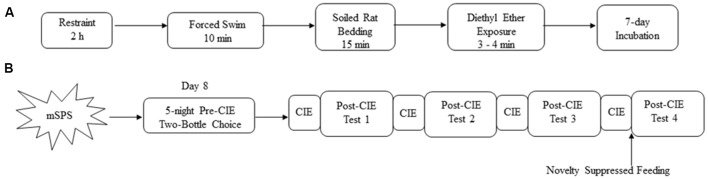
Schematic overview of **(A)** the mouse single-prolonged stress (mSPS) paradigm, **(B)** mSPS, and chronic intermittent ethanol (CIE) exposure with two-bottle choice drinking test. After the mSPS paradigm, mice were exposed to two-bottle choice for five nights. Mice were then exposed to CIE exposure every other week, with a Post-CIE drinking test in between the weeks of CIE exposure.

Adult male mice (10–12 weeks old) were exposed to mSPS which consisted of four consecutive stressors preceding a 7-day incubation period (see Figure 1A), as described previously (Matchynski-Franks et al., 2016; Perrine et al., 2016). Mice were restrained for 2 h in 50 ml conical tubes, with a screw top and holes along the tube for adequate air exchange. After 2 h of restraint, mice were immediately exposed to a 10-min group (*n* = 4–5 mice/group) forced to swim in 26–28°C water in a 4-L glass beaker. Mice were then towel-dried and returned to their home cages where they were exposed to a beaker filled with soiled rat bedding, a predator scent, for 15 min. Immediately after soiled rat bedding exposure, mice were placed in a clean microisolator polycarbonate cage with a cage lid. Cotton balls saturated with diethyl ether anhydrous were gradually placed in the cage at 1-min intervals until mice lost consciousness, which was verified by the toe-pinch method. Control mice (non-mSPS) were handled, weighed, and housed in another room during the mSPS procedure, experiencing no mSPS exposure. After mSPS exposure, both mSPS and control animals were housed individually in clean cages with fresh bedding and left undisturbed for 7 days with *ad libitum* access to food and water and daily health monitoring.

### Limited Access Two-Bottle Choice and Chronic Intermittent Ethanol (CIE) Vapor Exposure

Figure 1B shows an overview of the experimental timeline. Stable baseline (Pre-CIE) ethanol intake was achieved beginning on day 8, after a 7-day undisturbed post-mSPS period, using a limited access two-bottle choice paradigm for five consecutive nights. Thirty minutes before the beginning of the dark cycle, mice were introduced to two 150-ml drinking bottles containing 15% v/v ethanol or tap water for 1 h with free access to food. The ethanol solution was prepared fresh daily with 100% ethanol solution and tap water. The positions of the two bottles were altered daily to avoid side preferences. The amount of ethanol consumed was recorded daily by subtracting the weight of the ethanol and water bottles before and after the 1-h ethanol and water consumption period. Ethanol consumption was calculated by converting the ethanol intake in ml (±0.1 ml) and body weight (±0.1 g) to g of ethanol intake/kg of body weight. Ethanol preference was calculated as a ratio of ethanol intake to total fluid consumed. Average ethanol intake and preference per animal were calculated based on average ethanol consumption and preference over the 5-night pre-CIE period.

Approximately 72 h after the last baseline ethanol intake session, mice were counterbalanced into Control-Air, Control-CIE, mSPS-Air, and mSPS-CIE groups according to their baseline ethanol intake levels. Mice, within their home cages, were placed in Plexiglas inhalation chambers (Plas Labs, Lansing, MI, USA) and exposed to ethanol vapor or air for 16 h followed by 8 h room air exposure (ethanol abstinence period) for four consecutive days, which was considered to be one cycle, based on published methods (Becker and Lopez, 2004; Anderson et al., 2016b). Ninety-five percent ethanol was mixed with air and vaporized at a rate of 10 l/min, and the ethanol concentrations in the chambers were monitored daily with a breathalyzer (BAC Track Select S80, San Francisco, CA, USA). Before placement into the ethanol or air chambers, mice were co-administered the alcohol dehydrogenase inhibitor pyrazole (Chem-Impex International, Wood Dale, IL, USA; 1 mM/kg) and ethanol (CIE exposure mice; 1.6 g/kg; 20% w/v) or saline (air exposure mice; 10 ml/kg) intraperitoneally to stabilize BECs and initiate ethanol intoxication, according to published methods (Becker and Lopez, 2004; Anderson et al., 2016b). BECs were measured once per cycle from tail blood samples. Blood samples were collected and centrifuged for 5 min at 2,000 *g*. Plasma was collected and measured using alcohol diagnostic reagents (Pointe Scientific, Canton, MI, USA Cat #: A7504). BEC levels above 175 mg/dl during each cycle indicated intoxication in C57Bl/6 mice (Becker and Lopez, 2004). Average BEC levels across four cycles were recorded. The CIE vapor or air exposure was repeated for 4 weekly cycles with 5-night two-bottle choice Post-CIE drinking sessions during intervening weeks.

### Novelty Suppressed Feeding (NSF)

Forty-eight hours after the last day of the 4th CIE cycle, mice were exposed to the NSF test (see Figure 1B). Sixty-four hours before the test, mice were given sweetened fruit cereal to be used in the NSF test in their home cages. Mice were then food, but not water, deprived for 48 h with 1 h free access to food every 24 h. On the testing day, a piece of sweetened fruit cereal was placed on a piece of filter paper in the center of an arena (62 cm × 62 cm × 36 cm). Mice were then placed in a random corner of the arena and latency to enter the arena center and feed was recorded in seconds. Mice that took longer than 600 s to take their first bite were eliminated from the test. After the first bite of the sweetened fruit cereal was consumed, mice were immediately removed from the arena and returned to their home cage, where they were offered a pre-weighed piece of sweetened fruit cereal for 5 min to determine home cage consumption. Percent of home cage sweetened fruit cereal consumption was calculated by subtracting the weight of sweetened fruit cereal left in the home cage from the original weight of the sweetened fruit cereal divided by the original weight of the sweetened fruit cereal, multiplied by 100%.

### Data Analysis and Statistics

Data calculations and statistical analyses were performed using MS Excel, GraphPad Prism 6 (San Diego, CA, USA), and Statistica 6.0 (Tulsa, OK, USA). Two mice were removed from the NSF analysis because one mouse’s latency to feed did not meet the 600 s or less criterion, and the other mouse datum was a statistical outlier, being more than two standard deviations away from the group mean. A student’s two-tailed *t*-test was used to analyze baseline ethanol consumption, baseline ethanol preference, and BEC values. For the Post-CIE ethanol consumption and ethanol preference results under various test weeks, a repeated measure of three-way ANOVA was used with mSPS, vapor exposure, and test week as factors, followed by Fisher’s LSD *post hoc* multiple comparisons tests, when appropriate. A two-way ANOVA was used to analyze the latency to feed values and the percentage of in-cage food consumption in the NSF test. All data are reported as mean ± SEM with p < 0.05 as the criterion for statistical significance.

## Results

**Figure 2:**
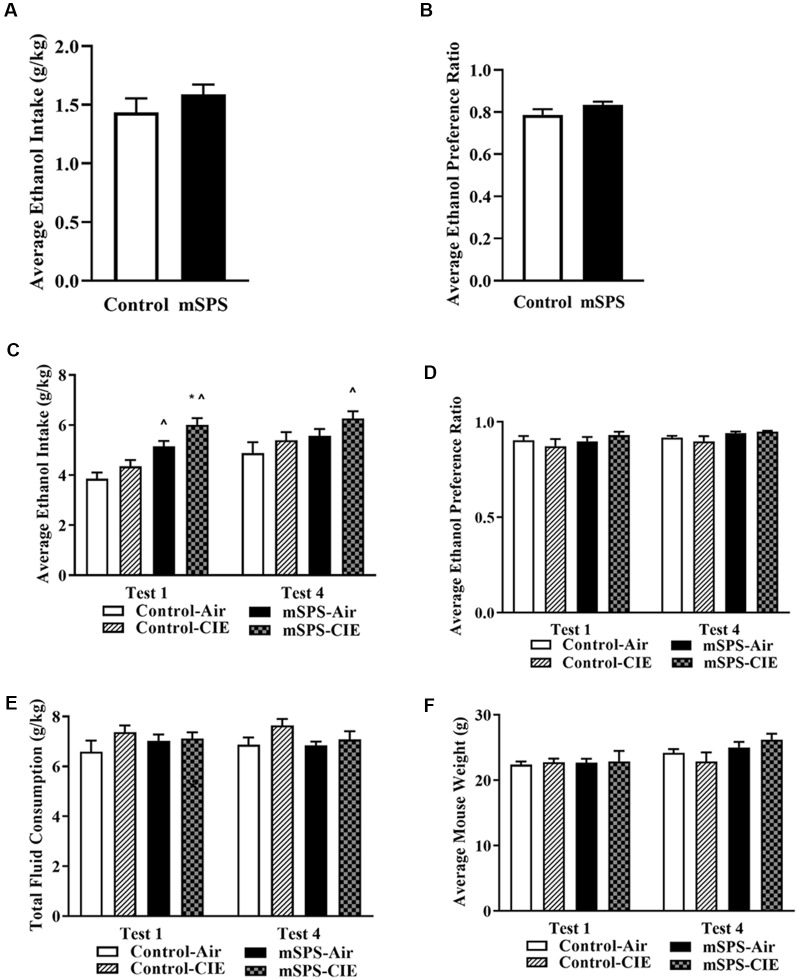
Average of Pre-CIE **(A)** ethanol intake and **(B)** ethanol preference on the 8th day after 7-day of incubation. A student’s two-tailed *t*-test revealed no significant impact on ethanol consumption and preference after mSPS (*n* = 14). However, a three-way ANOVA with repeated measures and *post hoc* Fisher-LSD test showed the results in **(C)** that vapor-induced ethanol intake was significantly increased in mSPS-CIE (*n* = 5) compared to Control-Air (*n* = 5; ^∧^*p* < 0.05) and Control-CIE mice (*n* = 4; *p < 0.05) in the 1st and 4th Post-CIE ethanol intake test sessions (Test 1 and 4). Mice exposed to mSPS and air control (mSPS-Air: *n* = 4) showed increased ethanol consumption in the 1st post-CIE ethanol intake session (Test 1; ^∧^*p* < 0.05) compared to Control-Air mice, but not in subsequent Post-CIE ethanol intake test sessions. The results in **(D)** showed no significant impact on ethanol preference among groups in either test session. A three-way ANOVA with repeated measures showed that neither the **(E)** average total fluid consumption among groups nor the **(F)** mouse body weights were affected in Test 1 and 4. Data are mean ± SEM.

mSPS exposure did not increase baseline Pre-CIE ethanol intake (student’s two-tailed *t*-test *t*_(26)_ = 1.04; *p* = 0.31; Figure 2A) or ethanol preference (*t*_(26)_ = 1.56; *p* = 0.13; Figure 2B). However, mSPS-CIE mice escalated their average ethanol consumption after their 1st (6.01 ± 0.27 g/kg) CIE cycle and sustained their average ethanol consumption at the 4th (6.26 ± 0.20 g/kg) CIE cycle. A three-way ANOVA with repeated measures revealed an mSPS effect (*F*_(1,14)_ = 17.9, *p* < 0.05), a CIE effect (*F*_(1,14)_ = 5.9; *p* < 0.05), and a week × mSPS interaction (*F*_(1,14)_ = 5.01; *p* < 0.05) on the average ethanol consumption (Figure 2C) after the 1st and 4th CIE cycle. A Fisher’s LSD *post hoc* comparison test revealed that the mSPS-CIE (*n* = 5) group significantly increased their vapor-induced ethanol consumption in Test 1 and continued to consume a similar amount of ethanol in Test 4 compared to the Control-Air (*n* = 5; *p* < 0.05), and the Control-CIE groups (*n* = 4; *p* < 0.05) in Test 1. mSPS-Air mice (*n* = 4; *p* < 0.05) also significantly increased their average ethanol consumption compared to the Control-Air group after the first cycle of air. Even though mSPS increased average ethanol consumption, it did not increase ethanol preference, which was approximately 92% (Figure 2D) after four cycles of CIE. For the average water consumption, a three-way ANOVA with repeated measures showed an mSPS-effect (*F*_(1,12)_ = 18.4; *p* < 0.05). A Fisher’s LSD *post hoc* comparison test showed that the mSPS-Air mice (2.32 ± 0.29 g/kg) and mSPS-CIE (2.08 ± 0.2 g/kg) consumed less water than the Control-CIE mice (3.29 ± 0.35 g/kg) in Test 1. In Test 4, only the mSPS-CIE mice (2.18 ± 0.20 g/kg) consumed less water than both Control-Air (3.00 ± 0.16 g/kg) and Control-CIE (3.16 ± 0.30 g/kg). For the average total fluid consumption, a three-way ANOVA with repeated measures (*F*_(1,12)_ = 0.041; *p* = 0.84) showed no significant differences among groups (Figure 2E). In addition, a three-way ANOVA with repeated measures (*F*_(1,14)_ = 2.02; *p* = 0.18) revealed no differences in body weights among groups (Figure 2F).

**Figure 3:**
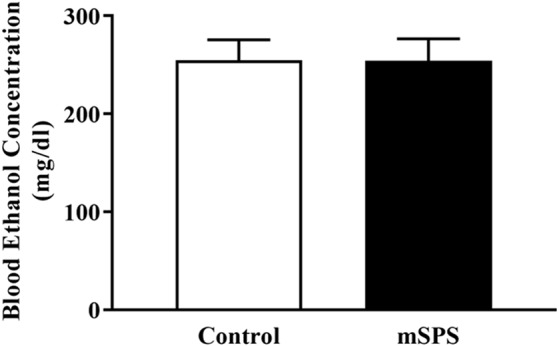
Average blood ethanol concentrations (BECs; mg/dl) across four cycles of CIE vapor exposure. Average BECs in mSPS-CIE mice (*n* = 5) over four cycles of CIE vapor exposure did not differ from the Control-CIE mice (*n* = 4). Data are mean ± SEM.

During each CIE cycle, BECs were evaluated in both the Control-CIE and mSPS-CIE groups. Figure 3 shows the average BEC levels across four cycles between the Control-CIE and mSPS-CIE groups. A student *t*-test revealed that there was no significant difference in BEC levels between Control-CIE (*n* = 4; 258 ± 17 mg/dl) and mSPS-CIE (*n* = 5; 262 ± 18 mg/dl) mice. BEC levels were undetectable in Control-Air and mSPS-Air mice (data not shown).

**Figure 4:**
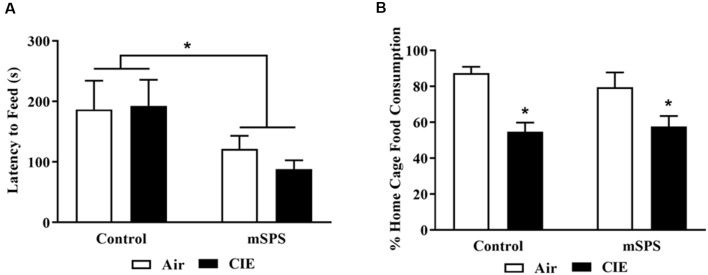
Average of **(A)** latency to feed during the novelty suppressed feeding (NSF) test and **(B)** percent sweetened fruit cereal consumed in the home cage, after the 4th cycle of CIE (before Test 4). mSPS increased impulsivity behavior **(A)**, and CIE exposure decreased home cage sweetened cereal intake in mice (*n* = 5–6; **B)**. A two-way ANOVA revealed that mSPS-exposed mice, either exposed to CIE or Air, had a shorter latency to take the first bite of a sweetened fruit cereal (*p < 0.05) compared to mSPS controls. A two-way ANOVA also revealed that CIE-exposed mice, either exposed to mSPS or Control (non-mSPS) conditions previously, consumed less sweetened cereal in their home cages compared to Air-exposed mice. Data are mean ± SEM.

NSF was performed 48 h after the last day of CIE exposure, before Test 4. Figure 4 shows the (Figure 4A) latency to feed and (Figure 4B) % consumption of sweetened cereal in the home cage during the NSF test. One mouse was eliminated because his latency to feed was over 600 s, and an outlier datum was also eliminated from the analysis. Results of the latency to feed showed a mSPS effect (two-way ANOVA; *n* = 12; *F*_(1,21)_ = 5.304; *p* < 0.05). mSPS-exposed mice (108 ± 14 s), either exposed to Air (*n* = 7) or CIE (*n* = 5), had a shorter latency to consume the first bite of sweetened fruit cereal in the arena compared to the Control (non-SPS) mice (189 ± 31 s). Results of the percent home cage cereal consumption revealed an ethanol vapor effect (two-way ANOVA; *n* = 11; *F*_(1,21)_ = 19.39; *p* < 0.05). Mice exposed to CIE (56 ± 4%), either Control (*n* = 6) or previously exposed to mSPS (*n* = 5), consumed less cereal in the home cage compared to mice exposed to Air (83 ± 4%).

## Discussion

The current study evaluated the effects of traumatic stress exposure, chronic alcohol exposure, and co-occurring of both exposures on behavioral outcomes including ethanol intake and preference, impulsivity, and anhedonia. This study hypothesized that mice exposed to mSPS would escalate their ethanol consumption and preference earlier after CIE and withdrawal and sustain their ethanol intake until the end of the study compared to controls. These results were predicted to be associated with increased impulsivity and anhedonia compared to controls. Results showed that mice exposed to mSPS did not escalate their Pre-CIE two-bottle choice ethanol intake. However, once mSPS-exposed mice were exposed to CIE, which included 8 h/day ethanol abstinence and another 81-h ethanol abstinence period between the CIE cycle and Post-CIE drinking session, their ethanol intake was significantly increased in Test 1 and sustained at Test 4 while their ethanol preference remained unchanged. Even though the ethanol intake was increased in mSPS-CIE mice, their BEC levels did not differ from the Control-CIE mice, indicating both groups were intoxicated during CIE exposure. Finally, mice exposed to mSPS had a shorter latency to feed on a palatable reward, sweetened fruit cereal, during the NSF test, and mice exposed to CIE consumed less sweetened fruit cereal in their home cage after the NSF test.

Previous studies from other laboratories have exposed mice to Pre-CIE drinking (two-bottle choice limited access) for 3–6 weeks to get a stable baseline of ethanol intake before stress and CIE (Anderson et al., 2016a,b; Lopez et al., 2016; Rodberg et al., 2017). Also, these studies showed that non-dependent mice exposed to different stressors did not escalate ethanol consumption if mice were exposed to a limited access drinking paradigm only (Anderson et al., 2016a; Lopez et al., 2016). Following this, our current study also showed that mice exposed to traumatic stress did not increase their baseline ethanol consumption, indicating that ethanol dependence does not develop during the baseline drinking session.

Effects of stress on ethanol consumption behaviors have provided variable results in animal models (Pohorecky, 1990; Sillaber and Henniger, 2004; Yang et al., 2008; Becker et al., 2011; Cozzoli et al., 2014; Lopez et al., 2016). Rodents’ drinking behaviors can be affected by different types of stress or durations of ethanol access. One study showed that restraint stress did not change ethanol consumption when mice had 24-h ethanol access (Yang et al., 2008), while another study found ethanol consumption to be decreased after restraint stress when mice had 2-h limited ethanol access (Cozzoli et al., 2014). Also, mice exposed to predator odor stress escalated their alcohol consumption 2 days after stress (Cozzoli et al., 2014). In a modified SPS study, rats that were exposed to traumatic stress had a higher alcohol preference score during a conditioned place preference (CPP) test compared to controls (Yu et al., 2016), which indicated that traumatic stress could cause an escalation of ethanol intake.

In comorbid stress and AUD study, mice escalated their ethanol consumption after exposure to CIE with forced swim stress but had no change in their ethanol consumption after CIE with social defeat stress exposure (Lopez et al., 2016). This study further demonstrated that mice exposed to forced swim stress once during the last cycle of CIE did not increase their ethanol consumption (Anderson et al., 2016a; Lopez et al., 2016). These studies underscored the importance of the experimental design of stress and alcohol exposure comorbidity (types and durations of stress, duration, and route of ethanol exposure). Our Post-CIE two-bottle choice limited access had comparable results. mSPS-CIE and mSPS-Air mice consumed a high amount of ethanol during the Post-CIE two-bottle choice session after the 1st CIE cycle, mainly due to stress-induced ethanol consumption. A ceiling effect likely prevented the mSPS-exposed group from further escalating their ethanol intake by Test 4 compared to Test 1, as their ethanol consumption was elevated earlier (Test 1) than that of the Control-Air and Control-CIE groups. At Test 4 (after the 4th cycle), only mSPS-CIE mice showed exacerbated ethanol intake with no sustained changes in any other testing group, which is consistent with the results from published studies (Anderson et al., 2016a; Lopez et al., 2016). The Control-CIE group demonstrated a gradual increase in ethanol intake, as it has been shown in published studies (Lopez et al., 2012; Anderson et al., 2016a). Use of a 5-days pre-CIE baseline drinking session in the present study, instead of a 6-week pre-CIE baseline drinking session used in the original paradigm (Becker and Lopez, 2004), could explain this gradual increase of ethanol intake observed in the Control-CIE group. Yet, a novel finding for the present Post-CIE drinking study is that mSPS, which consists of a variety of stressors, had a rapid and protracted effect on ethanol consumption behavior, as seen in Test 1 and 4. Mice were exposed to mSPS 8 weeks before the Test 4 drinking session, and mice were not exposed to another mSPS during this period of eight-weeks. Therefore, mSPS-CIE comorbidity has a unique effect that caused mice to escalate their ethanol intake, similar to the increased ethanol intake phenomenon reported in humans with PTSD and AUD (Brown et al., 1999; Ouimette et al., 1999; Berenz et al., 2017). Even though mSPS-CIE mice escalated their ethanol intake in Test 4, mice exposed to either Control (non-mSPS) or mSPS had similar BEC levels. This result indicates that both Control-CIE and mSPS-CIE groups reached the critical threshold of intoxication (≥175 mg/dl) that is a criterion for the CIE animal model (Becker and Lopez, 2004). Both Pre-CIE and Post-CIE ethanol preference showed no difference among treatment groups, suggesting that ethanol intake may have produced a ceiling effect in our mice, which could obscure changes in mSPS-induced ethanol preference.

Clinical studies have shown that impulsive behavior is highly correlated with PTSD (Garfinkel et al., 2014; Armour et al., 2016; Contractor et al., 2016; Roley et al., 2017) and AUD (Dick et al., 2010) alone, as well as with the combination of PTSD and substance use disorder (Weiss et al., 2017). The NSF test is commonly used to measure novelty-induced hyponeophagia (Samuels and Hen, 2011). This test has been used to examine anxiety-like behavior after alcohol withdrawal (Pang et al., 2013; Holleran et al., 2016; Jury et al., 2017; Sidhu et al., 2018), as well as impulsive behavior (Bevilacqua et al., 2010; Angoa-Pérez et al., 2012) in mice. In impulsivity studies, mice had a shorter latency to feed in a novel environment (Bevilacqua et al., 2010; Angoa-Pérez et al., 2012), whereas, in ethanol-induced anxiety studies, mice had a longer latency to feed in a novel environment (Pang et al., 2013; Holleran et al., 2016; Jury et al., 2017; Sidhu et al., 2018). Also, anxiety-like behavior has been observed in CIE-exposed mice using both the marble-burying and the NSF tests (Rose et al., 2016; Jury et al., 2017; Sidhu et al., 2018). However, unlike the previous studies from others (Jury et al., 2017; Sidhu et al., 2018), our CIE-exposed mice did not show anxiety-like behavior, as they did not have a longer latency to feed in a novel environment. Our contradicting results could be due to differences in our CIE paradigm before the NSF test. Our CIE paradigm before the NSF test consisted of four cycles of CIE with a two-bottle choice limited drinking test during intervening weeks, whereas other studies used repeated CIE with no intervening drinking sessions. An extra two-bottle choice limited access drinking test in between CIE exposure could account for our NSF results differing from those of others. In our study, mSPS-exposed mice had a shorter latency to feed in a novel environment after both Air and CIE vapor exposures, which could be interpreted as impulsivity-like behavior, based on the findings from published studies (Bevilacqua et al., 2010; Angoa-Pérez et al., 2012). Additional studies have reported that C57/Bl6 mice show resilience to anxiety-like behaviors, with results being dependent on the ethanol intake regimen (Cox et al., 2013), which could explain why we did not see anxiety-like behavior on CIE-exposed mice.

Another behavior related to negative affect that is associated with AUD and PTSD is anhedonia, which can be reflected in the NSF test. According to the DSM-5, anhedonia is one of the symptoms associated with PTSD, as well as ethanol withdrawal (Becker, 2008; American Psychiatric Association, 2013). Mice exposed to a 6-week two-bottle choice drinking session followed by 2-week ethanol abstinence showed a decrease in saccharin consumption during a saccharin preference test, which suggests that mice showed anhedonia-like behavior after ethanol withdrawal (Pang et al., 2013). Furthermore, rats exposed to SPS consumed less saccharin during a saccharin preference test, consumed less cocaine during cocaine self-administration, and had a lower cocaine preference score during a CPP test, which indicates anhedonia-like behavior (Enman et al., 2015). In the current study, mice exposed to repeated CIE vapor exposure, with prior exposure to either Control or mSPS conditions, consumed less sweetened fruit cereal in their home cage after the NSF test, indicating anhedonia-like behavior. These results parallel the results of the saccharin preference test study (Enman et al., 2015). One would expect that mSPS-Air mice would have anhedonia-like behavior, as was seen in the SPS-cocaine rat study (Enman et al., 2015). However, the SPS effect may resolve 2 weeks after the exposure in the absence of additional insults, such as cocaine or ethanol exposure, within the 2 weeks (Liberzon et al., 1997, 1999; Feng et al., 2015).

In conclusion, this study examined the effects of traumatic stress and ethanol exposure on ethanol intake and negative affect behaviors such as impulsivity and anhedonia. The combined exposure produced a protracted increase in dependence-induced ethanol intake. Also, traumatic stress exposure in mice caused impulsivity-like behavior, and repeated CIE vapor exposure resulted in anhedonia-like behavior. Future studies will examine the connections between these behavioral outcomes and the molecular mechanisms in the brain after PTSD-AUD exposure. The cannabinoid (CB) system is one of the systems that could be affected by the comorbidity of PTSD and AUD, as previously reported by our lab (Matchynski-Franks et al., 2016). CB signaling is known to modulate the activation of stress responses (Crowe et al., 2014). Besides, cannabinoid 1 (CB1) receptors and the endocannabinoids play an important role in the motivation and reinforcement of ethanol and ethanol withdrawal (Varodayan et al., 2017). For example, CB1 receptor expression and function were downregulated while the ethanol consumption was increased after mice were exposed to CIE for two cycles (DePoy et al., 2013). However, the mechanisms by which the comorbidity of PTSD and AUD affects CB function are not yet known. Therefore, it is important to examine the disruption of CB regulation of PTSD and AUD comorbidity which could cause negative behavioral outcomes such as those found in this study.

## Data Availability Statement

The raw data supporting the conclusions of this article will be made available by the authors, without undue reservation.

## Ethics Statement

The animal study was reviewed and approved by Wayne State University Institutional Animal Care and Use Committee.

## Author Contributions

VP designed the experiments, performed the experiments, analyzed the data, and drafted the manuscript. SL performed the experiments. SP reviewed the data and interpreted the results. AC designed the experiments, reviewed the data, and interpreted the results. All authors provided critical inputs and revisions on the manuscript.

## Conflict of Interest

The authors declare that the research was conducted in the absence of any commercial or financial relationships that could be construed as a potential conflict of interest.

## Abbreviations

AUD: alcohol use disorder
BEC: blood ethanol concentration
CB: cannabinoid
CIE: chronic intermittent ethanol
mSPS: mouse single-prolonged stress
NSF: novelty suppressed feeding
PTSD: post-traumatic stress disorder
WSU: Wayne State University.
